# Long Non-coding RNA N1LR Protects Against Myocardial Ischemic/Reperfusion Injury Through Regulating the TGF-β Signaling Pathway

**DOI:** 10.3389/fcvm.2021.654969

**Published:** 2021-08-13

**Authors:** Lin Du, Jie Chen, Yong Wu, Guangwei Xia, Mingxing Chen, Pei Zhao, Yao Wang, Deshan Yao, Fan Liu, Lina Zhang, Xue Wang, Yi Yang, Liansheng Wang

**Affiliations:** ^1^Department of Cardiology, The First Affiliated Hospital of Nanjing Medical University, Nanjing Medical University, Nanjing, China; ^2^Department of Cardiology, Affiliated Hospital of Yangzhou University, Yangzhou University, Yangzhou, China; ^3^Department of Gastroenterology, Northern Jiangsu Province People's Hospital, Yangzhou University, Yangzhou, China

**Keywords:** acute myocardial infarction, LncRNA N1LR, ischemic reperfusion injury, cardiomyocytes, TGF-β pathway

## Abstract

Long non-coding RNAs (lncRNAs) have been shown to play critical roles in various cell biological processes. However, the mechanism of lncRNAs in acute myocardial infarction (AMI) is not fully understood. Previous studies showed that lncRNA N1LR was down-regulated in ischemic cerebral stroke and its up-regulation was protective. The current study was designed to assess the protective effect of N1LR and further to explore potential mechanisms of N1LR in ischemic/reperfusion (I/R) injury after AMI. Male C57BL/6J mice and H9c2 cardiomyocytes were selected to construct *in vivo* and *in vitro* pathological models. In H9c2 cell line, N1LR expression was markedly decreased after H_2_O_2_ and CoCl_2_ treatments and N1LR overexpression alleviated apoptosis, inflammation reaction, and LDH release in cardiomyocytes treated with H_2_O_2_ and CoCl_2_. Mouse *in vivo* study showed that overexpression of N1LR enhanced cardiac function and suppressed inflammatory response and fibrosis. Mechanistically, we found that the expression of transforming growth factor (TGF)-β1 and smads were significantly decreased in the N1LR overexpression group exposed to H_2_O_2_. In a summary, our study indicated that N1LR can act as a protective factor against cardiac ischemic-reperfusion injury through regulating the TGF-β/Smads signaling pathway.

## Introduction

Acute myocardial infarction (AMI), characterized by coronary artery occlusion and myocardial cell necrosis, is the leading cause of death in patients with heart disease (1, 2). In AMI patients, although an early re-established blood flow inside the occluded coronary was essential to protect ischemic cardiomyocytes, this reperfusion can aggravate myocardial cell injury due to artery vascular endothelial cells malfunction and activation of several inflammatory factors (3, 4). It was reported that reperfusion injury can count up to 40% of myocardial cell necrosis and reduce the therapeutic efficacy of active reperfusion therapy (5, 6). Currently, there is a general consensus that myocardial I/R injury is the main cause of cardiac cell death and cardiac dysfunction (4).

The underlying molecular mechanism of I/R injury was complex and multifactorial in which excessive inflammation and apoptosis play the essential roles in the initiation and development of I/R (7). Transforming growth factor-β1 (TGF-β1) is the most critical isotype of TGF-β, and participate in inflammation, apoptosis, and tissue repair (8). Furthermore, a close and solid relationship between the TGF signaling pathway and I/R was found and reported (9). Smad2 and Smad3 are the two major downstream regulators of TGF-β1 signaling, TGF-β1-induced phosphorylation of Smad2 and Smad3 promote cell inflammation and apoptosis and tissue fibrosis (10). Other studies demonstrated that TGF-β/Smads are involved in myocardial pathological process (11). Therefore, TGF-β/Smads signaling pathway might be a potential therapeutic strategy for heart diseases.

In the last decade, non-coding RNAs (ncRNAs), accounting for ~98% of human genes, have emerged as a hot spot for scientific research (12). Tremendous literature has indicated that ncRNAs play an important role in cell growth, differentiation, immunity and apoptosis (13, 14). Long non-coding RNAs (lncRNA) are a class of lncRNA ribonucleic acid sequences larger than 200nt in length. Previous reports have shown that lncRNAs regulate protein-coding genes at transcriptional and post-transcriptional levels in coronary heart disease (CAD) (15). Furthermore, LncRNA has been reported involved in the molecular mechanism of myocardial I/R injury (16). LncRNA-N1LR, an I/R induced lncRNA, was initially found to be significantly down-regulated in the cerebral I/R rat model. It has shown that lncRNA-N1LR was involved in cell death, angiogenesis and inflammation during ischemic stroke. The infarct size increased significantly with the decrease of N1LR. In addition, N1LR up-regulation can provide neuroprotection against ischemic stroke *via* inhibiting p53 expression (17). All this indicates that lncRNA-N1LR could have a strong protective effect in I/R injury and its potential mechanism is worth to be explored.

In the present study, we aim to test the effect of N1LR on cardiac I/R injury *in vitro* and *in vivo*. Moreover, the underlying mechanism was investigated in the study. We found the N1LR attenuated inflammation and apoptosis of H9c2 cells treated with hypoxia and improved cardiac function in mice. And TGF-β/Smads signaling pathway is one the main mechanisms for N1LR myocardiac protective effects. Taken together, this study provides a potential therapeutic target for I/R-induced cardiac injury.

## Materials and Methods

### Cell Culture and Treatment

The H9c2 cell line derived from rat ventricle was purchased from the Cell Bank of the Chinese Academy of Sciences, Shanghai Institutes for Biological Sciences (Shanghai, China) and was cultured in Dulbecco's modified Eagle medium (DMEM, Thermo Fisher Scientific, Waltham, MA, USA) supplemented with 10% fetal bovine serum (FBS Invitrogen, Gibco, USA) plus 100 mg/ml penicillin/streptomycin (Gibco, CA, USA) at 37°C in the incubator with 95% air and 5% CO_2_.H9c2 cells from different groups were then plated in 6-well plates (3 × 10^5^ per well) and treated with 100 μM H_2_O_2_ for 5h or 800 μM CoCl_2_ for 20h (Both purchased from Tianjin Kemiou Chemical Reagent Co., Ltd, Tianjin, China) in serum-free DMEM to induce apoptosis and inflammatory response. Treated cells were collected for further experiments.

### Adenoviral Transfection

Cardiomyocyte specific vector AAV9-N1LR (AAV9-CTnT-N1LR), Cardiomyocyte specific overexpression vector Adenoviral5-N1LR (Ad5-CTnT- N1LR) and their corresponding control vector (AAV9-CTnT-Control and Ad5-CTnT-Control)were purchased from Shanghai OOBIO Biotechnology Company (Shanghai, China). For *vitro* experiments, 36h after infection with Ad5-CTnT-N1LR or Ad5-CTnT-Control at a multiplicity of infection (MOI) of 25, cells were treated with 100 μM H_2_O_2_ for 5h or 800 μM CoCl_2_ for 12h in serum-free DMEM. For *vivo* experiments, each mouse was injected with either AAV9-CTnT-N1LR or AAV9-CTnT-Control (1 × 10^12^ v.g/ml) *via* tail vein two weeks before I/R operation. Transfection efficiencies were validated by quantitative real-time PCR.

### Mouse Model of I/R Injury

The male C57BL/6J mice (6–8 weeks) were purchased from the experimental animal center of Yangzhou University. The study protocol was approved by the ethics committee of the Affiliated Hospital of Yangzhou University and was consistent with the Guide for the Care and Use of Laboratory Animals published by the National Institutes of Health (NIH publication 96-01).

Mice were randomly divided into four groups: NC, N1LR, I/R, I/R+N1LR. The myocardial I/R mouse model was established as previously described (18). Anesthetized (xylazine, 5 mg/kg i.p.) mouse was placed on the operating table, and then was intubated and connected to a rodent ventilator (ALCOTT BIOTECH CO, Shanghai, China). The heart was exposed through a left lateral thoracotomy and the left anterior descending artery (LAD) was ligated by 8-0 nylon suture with a slipknot. After that, the thorax was closed and the mouse was placed on a heating pad to keep the body warm. ST-segment elevation in ECG and regional blanching of the left ventricle were observed to ensure successful myocardial ischemia. After 60-min ischemia, the ligated slipknot was removed to perform consequential 2h or 4 weeks of reperfusion.

### Enzyme-Linked Immunosorbent Assay (ELISA)

After various treatments, the blood samples and supernatant of cell culture were collected and then centrifugated to remove cellular debris, the IL-6, IL-1β, and TNF-α levels were quantified using ELISA assay (Elabscience, Wuhan, China) according to the manufacturer's protocol. Analysis of optical density was performed in a standard microplate reader. The concentrations of IL-6, IL-1β, and TNF-α were calculated based on the standard curve.

### Cell Death Analysis and Lactate Dehydrogenase (LDH) Release Assay

Cells transfected with different adenoviral vectors were plated in 6-well plates (3 × 10^5^ per well). After adhesion, cells were treated with either H_2_O_2_ or CoCl_2_ to induce apoptosis and inflammatory response. To assess the cell death rates, the cells were collected and stained using Trypan Blue (Solarbio, Beijing, China) according to the protocol. The stained cells were observed under a light microscope at × 10 magnification, the numbers of Trypan Blue-positive and Trypan Blue negative were counted using a hemocytometer. Cell death rates (%) were calculated as (number of dead cells/total number of cells) ×100%.

Lactate dehydrogenase (LDH) was a marker of cell injury. The level of LDH released in the serum and cell culture supernatant was evaluated using LDH Release Assay Kit (Beyotime, Nantong, China) according to the manufacturer's protocols. The absorbance of the samples was measured at 490 nm using a microplate reader.

### Quantitative Real-Time PCR Analysis

Total RNAs from treated cells or heart samples were extracted using Trizol and treated with DNAase I to remove genomic DNA, and then purified with an RNA purification kit (Invitrogen, USA). cDNA was synthesized using Script cDNA Synthesis Kit (Bio-Rad, Hercules, CA). ABI 7900HT Fast Real-Time PCR System (Applied Biosystems) was used to quantify the expressions of genes. GAPDH was used as an internal normalized reference. The sequences of primers for each gene were shown in Table 1. The 2^−Δ*ΔCt*^ method was used to calculate the relative expression levels of different genes.

**Table 1:** Sequence of primers.

**Genes**	**Forward primer**	**Reverse primer**
GAPDH	CCTTCCGTGTTCCTACCCC	GCCCAAGATGCCCTTCAGT
N1LR	CGCGCTGCCATGACTGACA	CCGCTCTGGTCGGCGTCCT
IL-1β	TCACAGCAGCACATCAACAA	TGTCCTCATCCTGGAAGGT
TNF-α	ACGGCATGGATCTCAAAGAC	GTGGGTGAGGAGCACGTAGT
IL-6	GACTGCGGCAGAATTGCTATC	CGGGCTAATTTCCGTTGCATA
TGF-β1	CGCGGAGATGGAAGCACCGC	CCGCTCACCAAAGCTAAGAC
Colla1	CGCAGCACGTAGCGCACATC	GCCTTTGTGAGCGAACCCGA
Col3a1	GGTTTCCGGGATTGAGGCTG	TGCCCGTCTAATGAATCGGG
a-SMA	TGGGGTACCGGGTATAATCC	ACTGAAGTACGGCCCGTTCA

### Terminal Deoxynucleotidyl Transferase-Mediated dUTP Nick-End-Labeling Assay

H9c2 Cells in different groups were fixed by 4% paraformaldehyde. Terminal deoxynucleotidyl transferase-mediated dUTP nick-end-labeling assay (TUNEL) kit (Zhongshan Biotechnology Co, Beijing, China) was used to evaluate apoptosis of cardiomyocytes according to the manufactory's instructions. Images (100 × ) were taken using a fluorescence microscope (Nikon, Eclipse Ti, Japan).

### Echocardiography

4 weeks after I/R operation, mice were anesthetized again with 1.5–2.0% isoflurane, transthoracic M-mode and Color Doppler mode echocardiograms were applied to evaluate the cardiac function using an ultra-high resolution small animal ultrasound Vevo 3100 Imaging System (VisualSonics, Fujifilm) with a 30 MHz transducer. The left ventricular ejection fraction (LVEF), fractional shortening (FS) Left ventricular internal diameter (LVID) and left ventricular posterior wall (LVPW) were calculated through Simpson's measurements.

### Heart Histological Analysis

At the end of reperfusion, the mice were anesthetized and body weight (BW) was weighted. Then the heart tissues were harvested and weighed (heart weight HW), To delineate the infarct size of the myocardium, the hearts were cut into 2 mm-slices and the slices were incubated in 1% triphenyl tetrazolium chloride (TTC, Sigma, USA) and fixed with 4% paraformaldehyde and photographed. For general histological analysis, routine hematoxylin and eosin (H&E) staining and Masson's trichrome staining (Solarbio, Beijing, China) were performed. Briefly, after dehydrating with ethanol series, clearing with xylene and mounting with neutral resins, images of the myocardial structure were captured by light microscope. Computer-assisted morphometric analysis of digitized images was performed with image analysis software (Motic Image Advanced, Xiamen, China). The scoring criteria for myocardial injury histoscore were evaluated by two pathologists under blind condition according to a scoring system as described previously (19), i.e.: (4) severe (necrosis with the diffuse inflammatory process), (3) moderate (extensive myofibrillar degeneration and/or diffuse inflammatory process), (2) mild (small multifocal degeneration with a slight degree of the inflammatory process), (1) minimum (focal myocytes damage), and (0) nil (no changes).

### Western Blot Analysis

H9c2 cells were lysed for 20 min on ice with RIPA lysis buffer (Solarbio, Beijing, China) containing protease inhibitors and protein phosphatase inhibitor (Both purchased from Roche, Switzerland). BCA protein assay kit (Thermo, Rockford, USA) was used to detect protein concentration. Protein samples were segregated by 10% SDS-PAGE and transferred to polyvinylidene difluoride (PVDF) membranes (EMD,Millipore, USA). After blocking with 5%BSA, the blots were probed with various primary antibodies including TGF-β1, T-Smad2, T-Smad3, p-Smad2, p-Smad3, and GAPDH (1:1000 dilution, All purchased from Cell Signaling Technology, Beverly, USA) at 4°C overnight and then incubated with anti-rabbit IgG H&L (HRP) secondary antibody (1:5000, CST, Beverly, USA) for 1h at room temperature. The bands on PVDF were visualized by use of a Super Western Sensitivity Chemiluminescence Detection System (Thermo, USA). Image-J was used to analyze the band intensities.

### Statistical Analysis

Statistical Analysis was performed using SPSS 17.0 software (SPSS Inc., Chicago, IL USA). Continuous variables were shown as “mean ± SD”. One-way analysis of Variance (ANOVA) was used to determine the significant differences of each group, and Student's T-test was used for comparisons between two groups. *P*-value < 0.05 was considered statistically significant.

## Results

### N1LR Attenuated the Death and LDH Release of Cardiomyocytes *in vitro*

**Figure 1:**
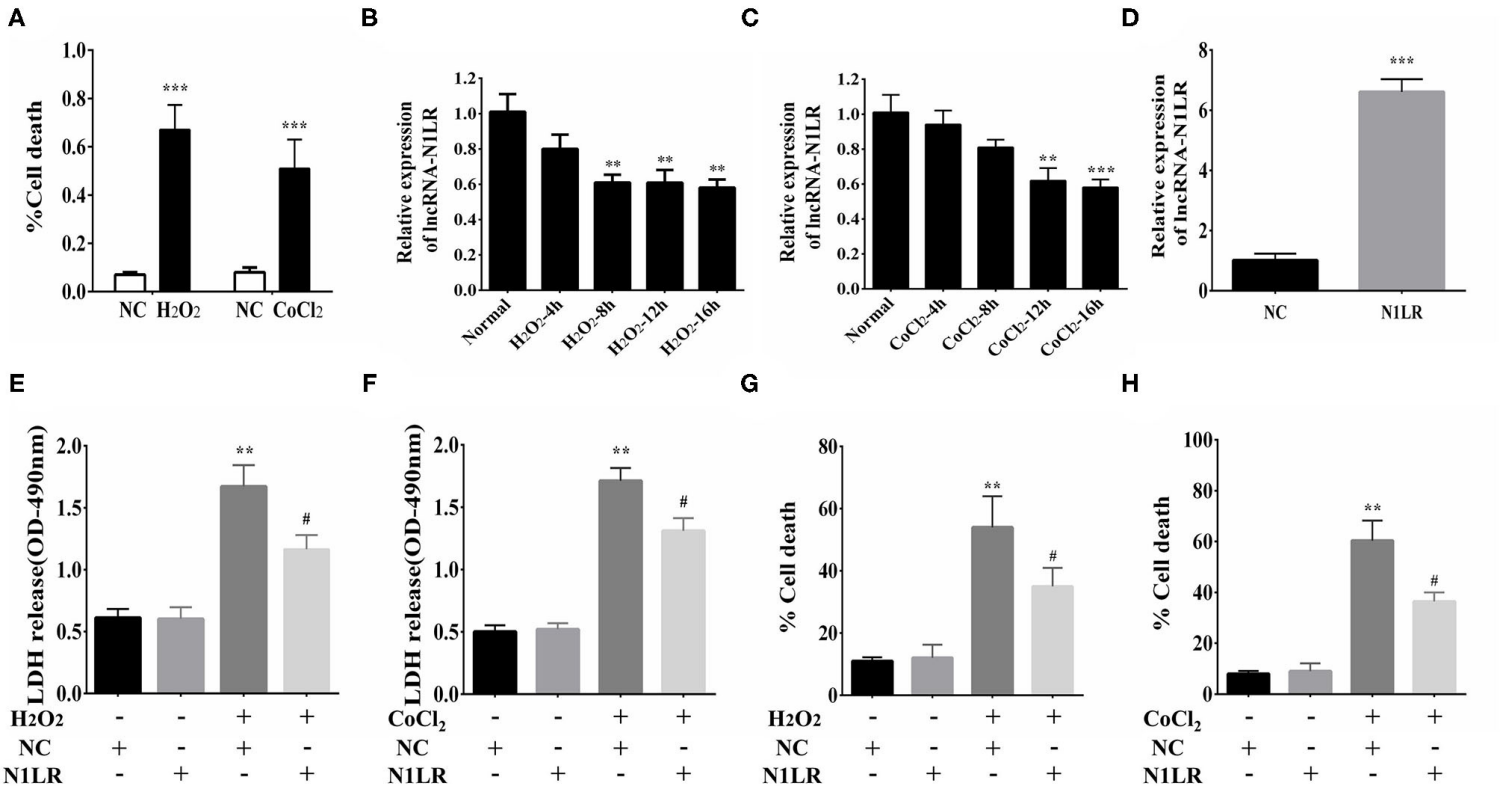
N1LR attenuated the cell death and LDH release of H9c2 cells. **(A)** Cell Death rates were measured to confirm the successful establishment of the hypoxia model. **(B)** The expression of N1LR was down-regulated with continuous exposure to hypoxia-induced by CoCl_2_. **(C)** The expression of N1LR was down-regulated with continuous exposure to hypoxia-induced by H_2_O_2_. **(D)** The overexpression of N1LR was verified through qRT-PCR. **(E)** N1LR reduced the release of LDH in H_2_O_2_-treated H9c2 cells. **(F)** N1LR reduced the release of LDH in CoCl_2_-treated H9c2 cells. **(G)** The H9c2 cell death was decreased in the N1LR+H_2_O_2_ group compared with the H_2_O_2_ group. **(H)** The H9c2 cell death was as well decreased in the N1LR+CoCl_2_ group compared with the CoCl_2_ group. **p < 0.01, ***p < 0.001 vs. NC group. ^#^*p* < 0.05 vs. H_2_O_2_+NC or CoCl_2_+NC group. Data were shown as means ± SD.

H_2_O_2_ treatment is well known for both cellular apoptosis and necrosis study (20). To determine the effect of N1LR on H9c2 cells, we first treated cells with H_2_O_2_ (100uM) or CoCl_2_(800uM) to induce cellular damage. The death rate of cardiomyocytes was increased after H_2_O_2_ and CoCl_2_ exposure, as shown by trypan blue staining (Figure 1A). Next, the expression of N1LR was detected using qRT-PCR. We found that the expression of N1LR was significantly reduced with continuous exposure to hypoxia in H_2_O_2_ and CoCl_2_ treated cardiomyocytes, respectively (Figures 1B,C). These results indicated that the level of N1LR was decreased during H_2_O_2_ or CoCl_2_-induced cell injury. Transfected with Ad5-CTnT-N1LR or vector, the expression levels of N1LR were about 6 times higher in the N1LR group compared with the NC group, indicating the transfection efficiency of lncRNA N1LR (Figure 1D). In addition, LDH release is related to cellular injury and was reduced in N1LR overexpressed group exposed to H_2_O_2_ and CoCl_2_ (Figures 1E,F). Moreover, it is worth noting that N1LR overexpressed reduced cell death compared with the NC group (Figures 1G,H). Thus, these results suggested that N1LR attenuated the cell death rate and LDH release of cardiomyocytes *in vitro*.

### N1LR Inhibits the Apoptosis and Inflammatory Response *in vitro*

**Figure 2:**
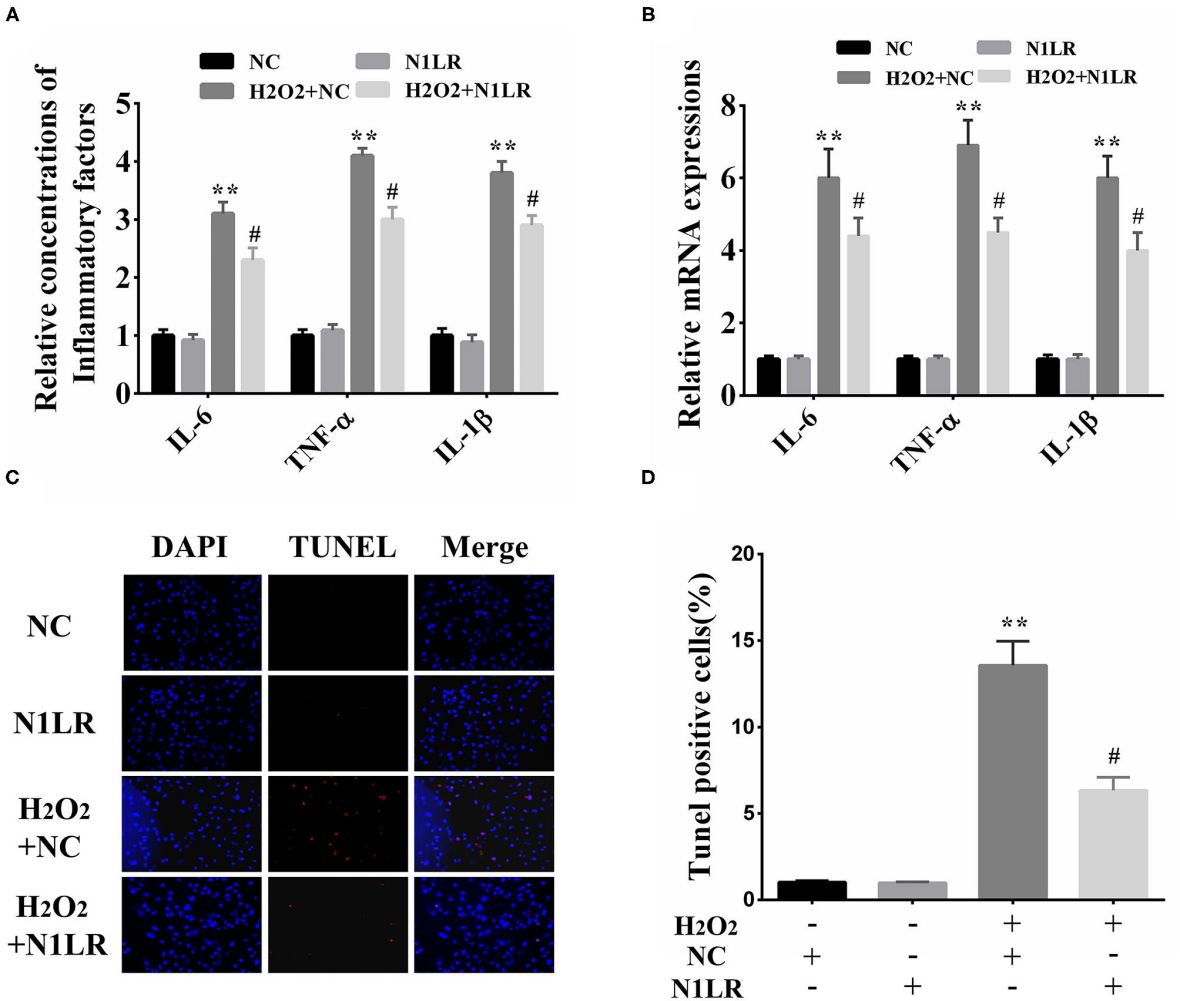
N1LR inhibited the apoptosis and inflammatory response *in vitro*. **(A)** Overexpression of N1LR decreased the levels of IL-6, TNF-α, and IL-1β in the supernatant measured by ELISA. **(B)** Overexpression of N1LR reduced the expressions of IL-6, TNF-α, and IL-1β mRNA. **(C)** A Tunnel assay was performed to measure the apoptotic rates. **(D)** The relative levels of TUNNEL-positive cells were measured *via* Image J software and apoptotic cells distinctly decreased in N1LR overexpressed group. **p < 0.01 vs. NC group, ^#^*p* < 0.05 vs. H_2_O_2_+NC group. Data were shown as means ± SD.

To investigate the specific effect of N1LR on H_2_O_2_-induced inflammation and apoptosis *in vitro*. We separately examined inflammatory-related factors (IL-6, TNF-α, and IL-1β) and apoptosis. ELISA results showed that the expression levels of IL-6, TNF-α, and IL-1β were reduced in N1LR overexpressed group (Figure 2A). Similar results were detected using qRT-PCR (Figure 2B). TUNEL assay was performed to assess the effect of N1LR on H_2_O_2_-induced apoptotic in H9c2 cells. We observed the tunnel-positive cells were distinctly decreased in the N1LR overexpressed group (Figures 2C,D). These results demonstrated N1LR alleviated the apoptosis and inflammatory response *in vitro*.

### N1LR Decreased Pro-inflammatory Factor Level and MI Infarction Area in the I/R Mice Model

**Figure 3:**
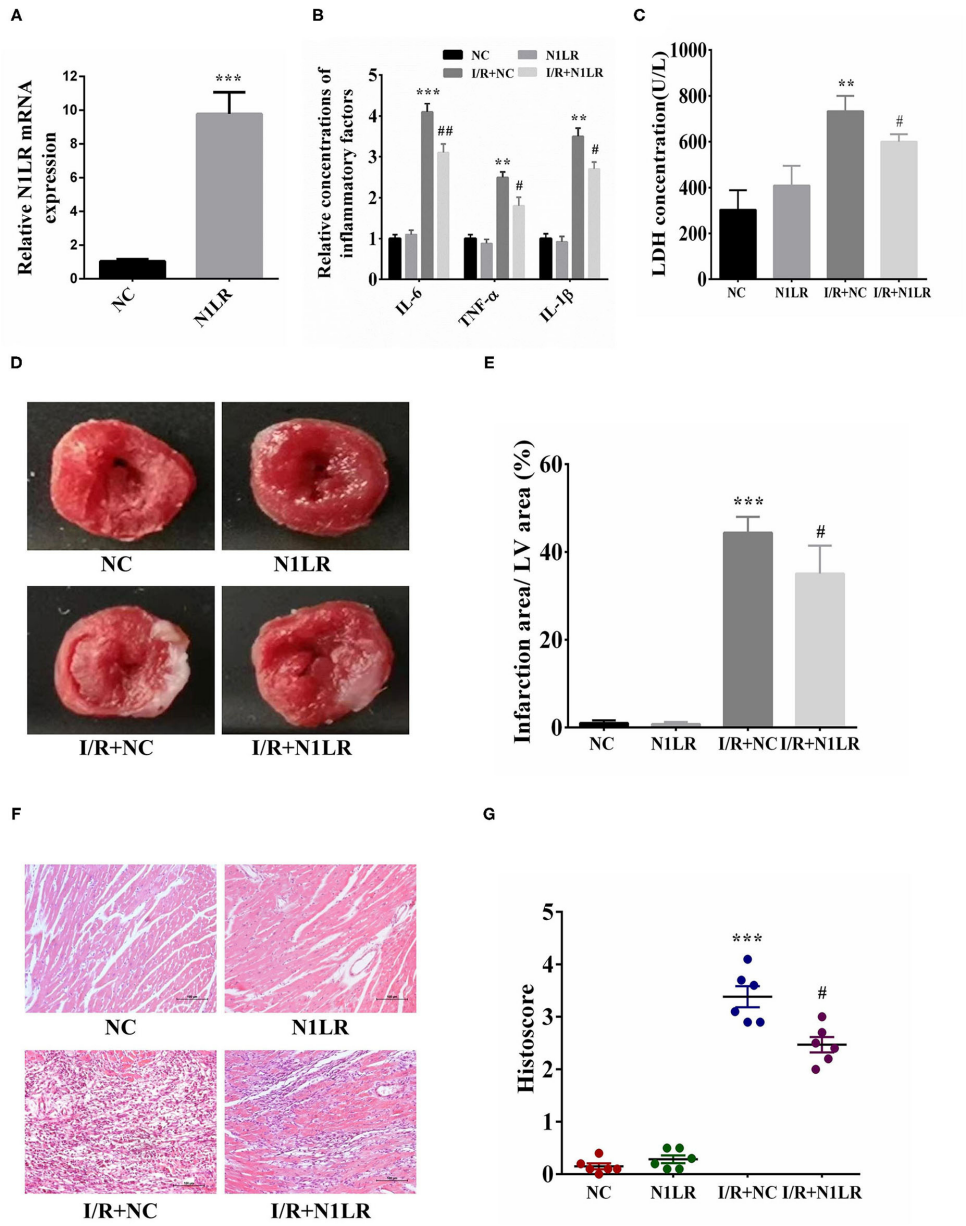
N1LR decreased pro-inflammatory factor level and infarction area in I/R mice model. **(A)** One day before the operation, N1LR mRNA expression in cardiac was verified by RT-qPCR. **(B)** N1LR decreased the IL-6, TNF-α, IL-1β levels in mouse serum. **(C)** N1LR reduced the levels of LDH in mouse serum. **(D)** Infarction area after myocardial I/R injury was detected by TTC staining. **(E)** N1LR decreased infarction area in the I/R mice model. **(F)** HE staining was performed to detect inflammatory infiltration ( × 200). **(G)** Histological scores were decreased in N1LR overexpressed group. Data are expressed as mean ± SD (*n* = 6–8). **p < 0.01, ***p < 0.001 vs. NC group. ^#^*p* < 0.05, ^*##*^*p* < 0.01 vs. I/R+NC group.

We then further testified N1LR's effects for I/R injury in mice. One day before I/R operation, N1LR mRNA expression in cardiac was verified by RT-qPCR. We can see the expression level of N1LR was about ten times higher than that in the NC group (Figure 3A). First, the inflammation effect was tested, after 2h of reperfusion the serum was collected for the detection of inflammatory markers. In the mice model, N1LR overexpression could significantly reduce inflammatory factors (IL-6, TNF-α, IL-1β) level which were caused by I/R jury (Figure 3B). LDH, as a cellular injury index, was also decreased by N1LR overexpression (Figure 3C). Those results implied that N1LR can reduce inflammation and attenuated the cardiomyocyte death caused by I/R jury *in vivo*. Meanwhile, for the histological analysis, TTC staining results showed that N1LR overexpression significantly diminished MI area (Figures 3D,E). In contrast, N1LR overexpression attenuated the morphology of cardiac muscle fibers as compared to the disarrangement in the NC group by using H & E staining (Figure 3F), this result was verified by the histological scores through ImageJ software for the N1LR cardiac protective function (Figure 3G). These observations revealed a cardioprotective role of N1LR overexpression in I/R induced myocardial injury *in vivo*.

### N1LR Ameliorated Fibrosis and Improved the Cardiac Function *in vivo*

**Figure 4:**
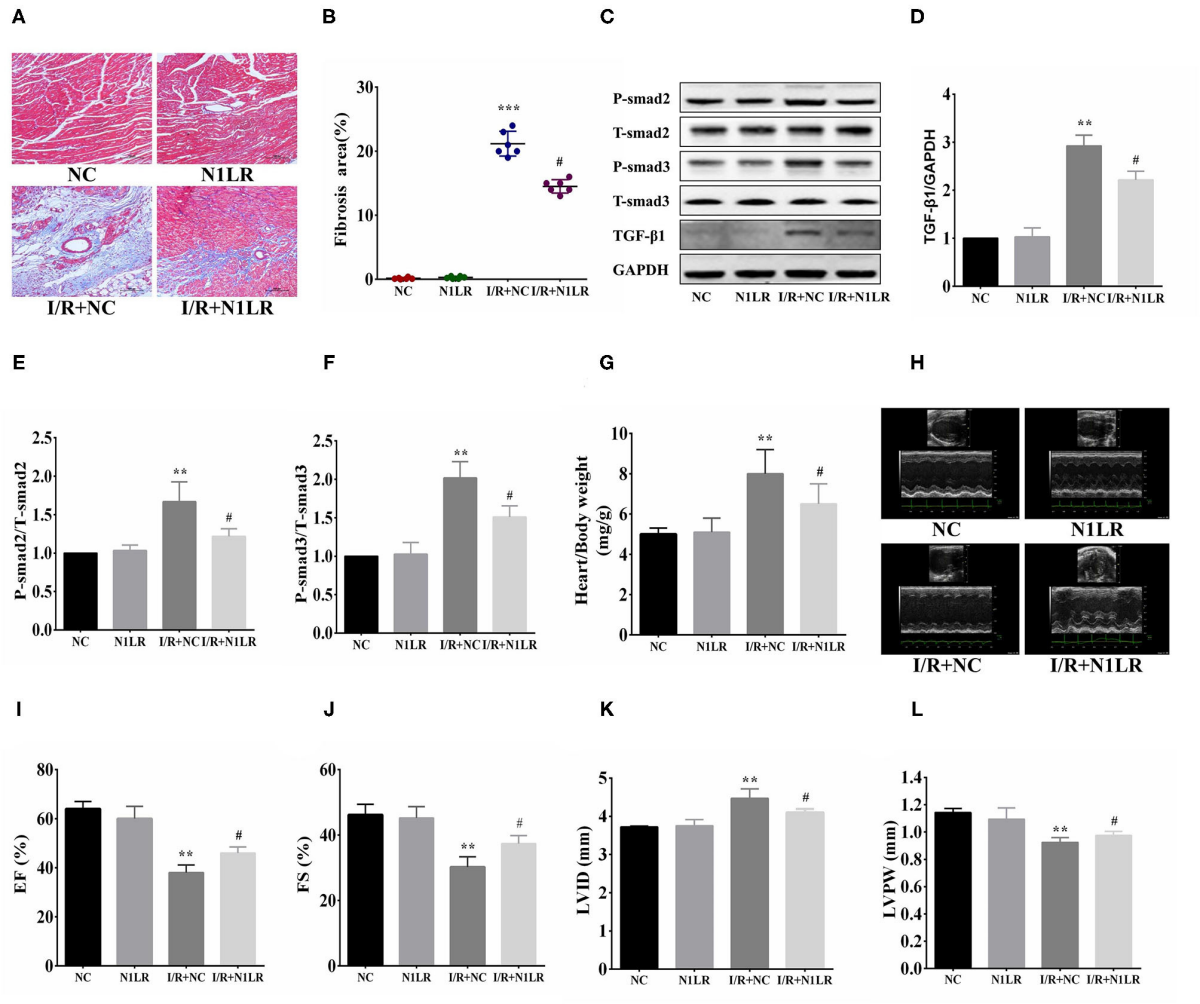
N1LR ameliorated fibrosis and improved cardiac function. **(A)** Masson Trichrome staining was performed to assess fibrosis ( ×200). **(B)** The fibrotic area was significantly reduced in N1LR overexpressed group. **(C)** TGF-β1, Smad2, P-Smad2, Smad3 and P-Smad3 were detected by Western blot. **(D–F)** Expressions of TGF-β1, P-Smad2 and P-Smad3 were markedly up-regulated in the I/R group. N1LR inhibited TGF-β1, P-smad2 and P-Smad3 expressions. The ratio of p-Smad2/T-smad2 and p-Smad3/T-smad3 were decreased compared to the I/R+NC group **(G)** Overexpression of N1LR decreased the ratio of heart weight to body weight. **(H)** Cardiac function was measured by echocardiography. **(I–L)** EF, FS, LVID and LVPW were improved in N1LR overexpressed group. Data are expressed as mean ± SEM. (*n* = 6–8). **p < 0.01, ***p < 0.001 vs. NC group, ^#^*p* < 0.05 vs. I/R+ NC group. Data are expressed as mean ± SD. (*n* = 6–8).

After inflammatory tests, we go further to examine the effect of N1LR overexpression on the consequent fibrosis 4 weeks after reperfusion. As shown by Masson Trichrome staining, the fibrosis area was significantly reduced in N1LR overexpressed group compared with the NC group in the mice model (Figures 4A,B). TGF-β1 is the most effective cytokine that induces the production of collagens in cardiac fibroblasts. Smad proteins, which are important and necessary for heart development and cardiomyocyte differentiation, are key downstream factors in TGF-β1 signaling pathway (21). TGF-β/Smads pathway plays a mechanical role in myocardial infarction and cardiac fibrosis (22). The effects of N1LR on the TGF-β1 and Smads proteins' expression in the cardiac tissue of I/R mice were investigated. Expressions of TGF-β1, T-Smad2, P-Smad2, T-Smad3, and P-Smad3 were detected by Western blot (Figure 4C). Treatment with N1LR could suppress expressions of TGF-β1, p-Smad2, and p-Smad3, the ratio of p-Smad2/T-smad2 and p-Smad3/T-smad3 were significantly lower than the I/R+NC group (Figures 4D–F). Worked as an indicator for cardiac hypertrophy, the ratio of heart weight to body weight is also reversed due to I/R jury by N1LR overexpression (Figure 4G). For the cardiac function study by echocardiography, both EF and FS were significantly reduced 4 weeks after myocardial I/R compared with baseline. LVID was significantly enlarged while LVPW was decreased after I/R practice. In contrast, cardiac dysfunction demonstrated a manifest improvement in the N1LR overexpressed group (Figures 4H–L). These results revealed that N1LR treatment can reduce fibrosis and hypertrophy and further improve cardiac function for a long-term benefit in the mice I/R model.

### N1LR Functioned *via* Repressing the TGF-β Signaling Pathway

**Figure 5:**
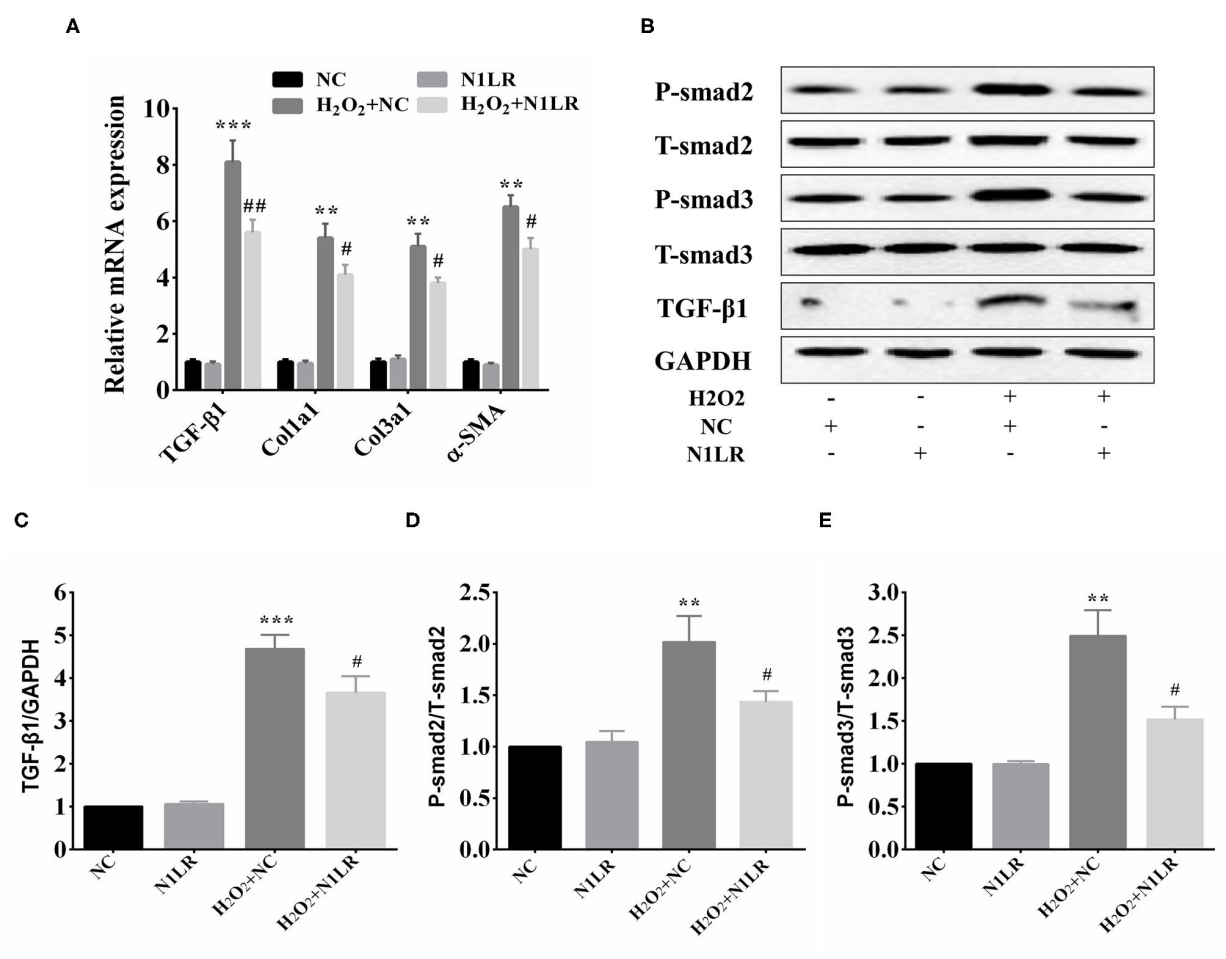
N1LR functioned *via* repressing TGF-β signaling pathway. **(A)** Levels of relevant markers TGF-β1, Col1a1, Col3a1, and α-SMA mRNA were measured by qRT-PCR. The expression of TGF-β1, Col1a1, Col3a1, and α-SMA were markedly decreased in N1LR overexpressed group compared with the H_2_O_2_+NC group. **(B)** Western blot analysis of TGF-β1, P-smad2 and P-smad3 in H_2_O_2_ treated H9c2 cells. **(C–E)** The relative expressions of TGF-β1, P-Smad2 and P-Smad3 were measured by using Image J software. Data are expressed as mean ± SD. **p < 0.01, ***p < 0.001 vs. NC group, ^#^*p* < 0.05, ^##^*p* < 0.01 vs. H_2_O_2_+ NC group.

The above results suggest that N1LR can ameliorate myocardial fibrosis after I/R injury by regulating the TGF-β1/Smad pathway. The effects of N1LR on the TGF-β1/Smad3 signaling pathway were further investigated in H_2_O_2_ treated H9c2 cells. According to the results of qRT-PCR, the mRNA expression levels of TGF-β1, Col1a1, Col3a1, and α-SMA were decreased in N1LR overexpressed group exposed to H_2_O_2_ (Figure 5A). After exposure to H_2_O_2_, the expressions of TGF-β1/smads also increased similarly to *in vivo* study. In brief, the protein expression levels of TGF-β1, P-Smad2, and P-Smad3 were manifestly increased and partially reversed in the N1LR overexpressed group, with unchanged total Smad2 and Smad3 (Figures 5B–E). Thus, our results suggested that lncRNA N1LR protected cardiomyocytes from H_2_O_2_**-**induced injury by repressing the TGF-β signaling pathway.

## Discussion

Immediate reperfusion could reduce the injury resulting from AMI, but also undermine cardiac function and the structure of myocardial cells (23, 24). The apoptosis and death of cardiomyocytes and the release of inflammation factors are boosted during I/R injury after AMI (25, 26). Myocardial inflammation is considered to be the secondary injury mechanism after I/R (27). Over the last decade, increasing reports have indicated that lncRNAs are involved in various human diseases, including AMI (15, 17, 28). Some lncRNAs are abnormally expressed in the development of CAD and implicated in cardiac pathophysiology, which suggesting lncRNAs serve as markers of pathogenesis and potential therapeutic targets (29, 30). For example, lncRNA-HOTAIR down-regulation worsens oxidative stress-induced cardiomyocyte injury through sponging miR-125 to inhibit the expression of MMP2 (28). Down-regulating lncRNA MALAT1 improves the outcomes of AMI through miR-320/PTEN axis (31). Specific up-regulation of miR-21 in rat hearts suppresses left ventricular remodeling and myocardial apoptosis induced by I/R injury (32). Studies show that lncRNA Novlnc6 is dramatically down-regulated in dilated cardiomyopathy, and Novlnc6 knockdown leads to the expression decreasing of BMP10 and Nkx2.5, which are two important regulators for cardiomyocytes maturation and differentiation (33). As a novel I/R-induced lncRNA, N1LR was demonstrated to have neuroprotective effects in ischemic mice (16). Overexpression of N1LR could effectively decreased infarct size and neurological deficit *in vivo*, while decreasing N1LR expression increased N2a cell apoptosis induced by Oxygen-glucose deprivation/reoxygenation(OGD/R) treatment *in vitro* (16).

It has been reported that oxidative stress and inflammation caused by hypoxia can induce cardiomyocyte injury and death. In the present study, we tested the effects of N1LR on cardiac I/R injury *in vivo* and H_2_O_2_-induced cell injury *in vitro*. We found that N1LR was down-regulated in H9c2 cells treated by H_2_O_2_ and CoCl_2_. There are many apoptotic cells but not dead cells in the treated cells. Overexpression of N1LR alleviated the H_2_O_2_-induced cell apoptosis, inflammation response, death, and LDH release in cellular experiments. In addition, N1LR improved cardiac function, ameliorated inflammation and fibrosis *in vivo*. These findings suggested that N1LR is an important regulator of hypoxia-resulted in injury for cardiomyocytes. We next explored the mechanism of N1LR overexpression in H9c2 cells under hypoxia induced by H_2_O_2_. Mechanistically, overexpression of N1LR significantly inhibited the expression of the TGF-β signaling pathway induced by I/R injury.

TGF-β1, a member of the cytokines family, plays an important role in physiological (like embryonic development, cell growth, and differentiation) and pathological processes (like inflammation, fibrosis, and apoptosis) (34, 35). Smads protein can transduce TGF-β1 family signal from the cell membrane receptor to the nucleus, thus forming TGF-β1/Smads signaling pathway to regulate myocardial fibrosis (36). Moreover, TGF-β1 is overexpressed in the heart in a high-cholesterol-fed porcine model of myocardial infarction, and its downstream Smad2 and Smad3 are activated, thereby increasing collagen synthesis and the levels of Col1a1, Col3a1, and α-SMA (37). In addition, the inflammatory response and apoptosis induced by I/R damage could be regulated by TGF-β1(38). These data support that the TGF-β1/Smads signaling pathway exerts an important effect on heart injury. *In vivo* study, the expression of TGF-β1, p-Smad2, and p-Smad3, were significantly lower than the I/R+NC group. *In vitro* study, the mRNA levels of TGF-β1, Col1a1, Col3a1, and α-SMA were decreased in N1LR overexpressed group exposed to H_2_O_2_, and the protein expressions of TGF-β1/smads are decreased similarly to *in vivo* study, suggesting that N1LR relies on the TGF-β1 pathway to reduce I/R-induced damage *in vivo* and *vitro*.

In conclusion, the present study suggested that N1LR overexpression represses the TGF-β1 pathway to inhibit H_2_O_2_-induced apoptosis, inflammatory response, and LDH release in cardiomyocytes after I/R-induced injury, all contributing to the improvement of cardiac function. Our findings provide new insight into the mechanism of I/R injury. The potential roles of N1LR in other cardiac myocyte disease models should be evaluated in subsequent studies.

## Data Availability Statement

The datasets presented in this study can be found in online repositories. The names of the repository/repositories and accession number(s) can be found in the article/supplementary material.

## Ethics Statement

The animal study was reviewed and approved by Athics committee of the Affiliated Hospital of Yangzhou University.

## Author Contributions

All authors contributed to the article and approved the submitted version.

## Conflict of Interest

The authors declare that the research was conducted in the absence of any commercial or financial relationships that could be construed as a potential conflict of interest.

## Publisher's Note

All claims expressed in this article are solely those of the authors and do not necessarily represent those of their affiliated organizations, or those of the publisher, the editors and the reviewers. Any product that may be evaluated in this article, or claim that may be made by its manufacturer, is not guaranteed or endorsed by the publisher.
